# Aortic Stiffness in Prediabetic Adults: Relationship to Insulin Resistance

**DOI:** 10.4021/jocmr2010.03.269w

**Published:** 2010-03-09

**Authors:** Hamdy Sliem, Gamela Nasr

**Affiliations:** a Departments of Internal Medicine, Suez Canal University, Ismailia, Egypt; b Departments of Cardiology, Suez Canal University, Ismailia, Egypt

## Abstract

**Background:**

A decrease in the compliance of the arterial system, termed arterial stiffness, results in increased cardiac workload. Several studies have shown that arterial stiffness is increased in individuals with type 2 diabetes. Also, insulin resistance is generally considered to be of major importance in the pathophysiology of type 2 diabetes mellitus given that glucose intolerance and insulin resistance precede the development of overt diabetes, these factors would be associated with arterial stiffness. This study was to evaluate the state of aortic elasticity in prediabetic adults in relation to insulin resistance.

**Methods:**

A case-control study was performed. A total of 113 consecutive adults with prediabetes were enrolled for the study, 32 adults had insulin resistance (group A) and 81 had insulin sensitive (group B). Forty-five healthy (with normal fasting glucose) adults matched for age and gender were considered as control. All were subjected to full medical history and clinical examination including blood pressure and body mass index. Biochemical studies including lipids profile, fasting glucose and homeostasis model assessment of insulin resistance (HOMA IR) test. Echocardiographic studies were done for assessment of the aortic stiffness index.

**Results:**

Significant increase in mean aortic stiffness index was seen in group A than group B. Stiffness was correlated with insulin resistance and the correlation appeared to be independent of glucose tolerance status and obesity. Similar correlations were observed with age, triglycerides and waist circumference.

**Conclusions:**

Prediabetic subjects have an aortic stiffness which represent pattern of cardiovascular risk factors. These changes are predominantly observed in prediabetic subjects with increased HOMA IR and visceral obesity independent of glucose levels.

**Keywords:**

Prediabetes; Insulin resistance; Aortic stiffness index

## Introduction

Diabetes mellitus, hypertension, dyslipidemia, obesity, and aging are associated with high risk of cardiovascular disease (CVD) as well as other clinical conditions [1]. The mechanisms through which cardiovascular risk is increased are partially understood. A decrease in the compliance of the central arterial system, termed arterial stiffness, results in increased cardiac workload [2]. As arterial changes can be detected before the appearance of clinically apparent vascular disease, arterial stiffness may act either as a marker for the development of future atherosclerotic disease, or may be more directly involved in the process of atherosclerosis [3].

A number of factors are believed to be responsible for arterial stiffness. These include decreased elastin and increased collagen in the arterial wall, abnormal endothelial regulation of arterial smooth muscle tone, and the accumulation of advanced glycosylation end products (AGE) leading to protein cross-linking [4-6].

Several studies have shown that arterial stiffness is increased in individuals with type 2 diabetes. Also, insulin resistance is generally considered to be of major importance in the pathophysiology of type 2 diabetes mellitus [7].

Given that glucose intolerance and insulin resistance precede the development of overt diabetes, these factors would be associated with arterial stiffness. To investigate this hypothesis, the current study was undertaken to evaluate the state of aortic elasticity in prediabetic adults in relation to insulin resistance.

## Patients and Methods

### Patient selection

A case-control study was performed. A total of 113 consecutive adults with prediabetes were enrolled for the study. All were recruited from the outpatient diabetes and general medicine clinics of Suez Canal University Hospital from May 2007 to October 2009. Thirty-two adults had insulin resistance (group A) and 81 had insulin sensitive (group B). Forty-five healthy (with normal fasting glucose) adults matched for age and gender were considered as a control group.

Exclusion criteria included the following, hypertension, diabetes mellitus, chronic kidney disease, CVD, severe obesity (body mass index > 40 kg/m^2^), heavy smokers, and aged adults (over 60 years old).

All groups were subjected to full medical history and clinical examination including blood pressure (BP), body mass index (BMI), systemic examination, biochemical and echocardiographic studies, anthropometric measures (weight in kilogram, height in centimeter, BMI, waist circumference). BMI was calculated as weight/height^2^ (kg/m^2^) and was used as an estimate of overall adiposity. Normal weight, overweight, and obesity were defined as a BMI less than 25, 25 to 29.9, and 30 or higher, respectively [8]. Waist circumference, a validated estimate of visceral adiposity, was measured to the nearest 0.5 cm. Central obesity is defined as waist circumference > 102 cm in males and > 88 cm in females [9].

Diabetes was diagnosed according to world health organization (WHO) criteria. Blood is drawn after fasting for eight hours. A fasting blood sugar level below 100 mg/dL is considered normal. A fasting blood sugar level between 100 and 126 mg/dL confirms the presence of prediabetes, and more than 126 mg/dL confirms the presence of diabetes in two separate occasions [10]. Homeostasis model assessment of insulin resistance (HOMA IR) was used as a measure of insulin resistance. It was assessed according to the level of fasting glucose and insulin which were measured with a dextran-charcoal radioimmunoassay. Serum intact pro-insulin was measured by using a highly specific, 2-site monoclonal antibody-based immunoradiometric assay [10, 11]. The formula for the HOMA IR model follows: HOMA IR = (Fasting insulin mU/mL X Fasting glucose mmol/L) / 22.5.

Subjects were divided by their insulin resistance status at baseline (HOMA IR above and below median of 3.0) to insulin resistant and insulin sensitive respectively. The median was based on the overall nondiabetic control group at baseline.

Aortic stiffness index (β) as a characteristic of aortic elasticity was evaluated from ascending aortic diameter. Aortic stiffness index ((β) was evaluated by means of transthoracic echocardiography by use of the formula: (β = ln (SBP/DBP) / (ΔD/ DD), where SBP and DBP are the systolic and diastolic blood pressures, DD is the diastolic aortic diameter,ΔD is the pulsatile change in aortic diameter (systolic diameter minus diastolic diameter) and ln is the natural logarithm [12].

### Ethical consideration

Informed consent was obtained from all the adults. The aim and the value of the work were explained in a simplified manner for them. There was no harm inflicted on them. On the contrary all had benefits of the follow-up and the final results of the study. The study was approved by the ethics committee of faculty of medicine, Suez Canal University.

### Statistical analysis

Data were presented in terms of mean and standard deviation (SD) of the mean, and percentages. Statistical analysis was carried out by a computer program (SPSS Ver. 11). Student-t test and correlation tests were used to evaluate the results. P value was set at less than 0.05 for statistically significant results and less than 0.0001 for highly significant results.

## Results

**Figure 1.**
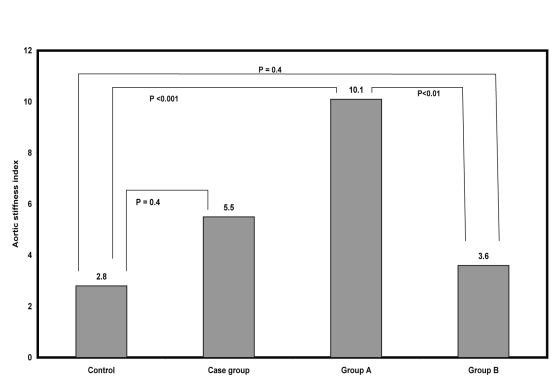
Mean Values of Aortic Stiffness Index in Case and Control Groups.

Baseline characteristics of 113 (60 males and 53 females) prediabetic adults, mean age 43.1 years, with and without insulin resistance versus 45 control (24 males and 21 females), mean age 44.4 years are shown in Table 1, 36.2% of the prediabetic adults had insulin resistance, 63.8% had insulin sensitive. No significant differences were observed between both prediabetic adult and control groups regarding BMI, waist circumference, blood pressure, and plasma lipids. Regarding aortic stiffness index, no significant difference was observed between either all prediabetic cases or group B and control group, while high significant differences were observed between group A and both group B and control (Fig 1). Except waist circumference all the mentioned parameters were nearly similar in both male and female prediabetic adults.

Correlation between aortic stiffness index and different variables of insulin resistant prediabetic group (group A) is shown in Table 2. There was statistically significant coefficient correlation with HOMA IR, waist circumference, triglycerides and age. No correlation was observed with fasting glucose, BMI, blood pressure and total lipids. Similarly, no correlations were found with overall prediabetic population variables.

**Table 1:** Clinical and Biochemical Studies of Both Case and Control Groups

Variable	Control Group N = 45	Prediabetic adults (Case) N = 113			P value		
		All N = 113	Group A N = 32	Group B N = 81	P^*^	P^**^	P^***^
Age	44.4 ± 7.8	45.1 ± 6.2	44.9 ± 7.1	45.3 ± 5.9	n.s	n.s	n.s
SBP	121.1 ± 8.2	122.2 ± 9.2	120.9 ± 7.9	120.1 ± 9.5	n.s	n.s	n.s
DBP	72.9 ± 7.6	73.9 ± 6.9	74.9 ± 6.5	73.5 ± 7.1	n.s	n.s	n.s
BMI	28.6 ± 5.7	28.4 ± 5.2	28.5 ± 5.2	28.4 ± 5.1	n.s	n.s	n.s
Waist circumference	100.3 ± 16.3	103 ± 17.2	107 ± 11.6	102.5 ± 18.2	n.s	n.s	n.s
Total lipids	501.1 ± 42.9	491 ± 31.9	479 ± 26.7	499.7 ± 36.4	n.s	n.s	n.s
Triglycerides	186.8 ± 15.6	187.7 ± 12.4	187 ± 14.2	188.5 ± 11.9	n.s	n.s	n.s
FBS	83.1 ± 7.2	114.5 ± 4.8	114.8 ± 4.6	115.7 ± 4.9	< 0.01	< 0.01	n.s
HOMA IR	2.9 ± 0.4	3.7 ± 2.5	7.5 ± 0.7	2.2 ± 0.4	< 0.01	n.s	n.s
Aortic stiffness index	2.8 ± 1.1	5.5 ± 3.1	10.2 ± 1.2	3.6 ± 0.7	< 0.001	n.s	n.s

Group A, prediabetic adult with insulin resistance. Group B, prediabetic adult with insulin sensitive. BMI, body mass index. SBP, systolic blood pressure. DBP, diastolic BP N=number of cases. n.s, non significant. FBS, fasting blood glucose. P^*^, comparison between group A and control. P^**^, comparison between group B and control. P^***^, comparison between all prediabetic adults and control group.

**Table 2:** Correlation Coefficient Between Aortic Stiffness Index and Different Variables in Group A

Variable	r value	P value
Age	0.81	< 0.01
Total cholesterol	0.13	n.s
Low density lipoprotein	0.24	n.s
Triglyceride	0.75	< 0.01
HOMA IR	0.91	< 0.001
Waist circumference	0.86	< 0.01
Total lipids	0.4	n.s
Systolic blood pressure	0.33	n.s
Diastolic blood pressure	0.36	n.s
High densitylipoprotein	0.21	n.s
Fasting blood glucose	0.19	n.s
Body mass index	0.16	n.s

## Discussion

Type 2 diabetes mellitus is associated with a marked increase in CVD. Increased risk factors for CVD before the onset of type 2 diabetes have been shown in several populations [1, 10, 13]. It is not known whether the increased atherogenicity of the prediabetic state is primarily due to increased insulin resistance or impaired blood glucose [14].

There has been much recent interest in the relationship between arterial stiffness and CVD. Pulse pressure, pulse wave velocity, and echographic surrogate measures of arterial stiffness, indicate that arterial stiffness increases both with age and in certain disease states that are themselves associated with increased cardiovascular risk, including hypertension, diabetes mellitus, hyperlipidemia, obesity, and end-stage renal failure [15-18].

However, the state of arterial elasticity in prediabetes has received little attention. We therefore studied prediabetic normotensive adults in whom the prevalence of insulin resistance and impaired glucose tolerance is high, to test the hypotheses that insulin resistance is associated with arterial stiffness and that this relationship is dependent or independent of glucose tolerance status.

The main finding of current study is that, insulin resistance is associated with arterial stiffness and that the association appears to be independent of glucose tolerance status and obesity. Hyperglycemia has been shown to lead to the formation of advanced glycosylation endproducts (AGEs). Therefore, it has been suggested that individuals with prediabetes may have increased central arterial stiffness due to prolonged exposure to elevated glucose levels [4, 6]. Results from the present study did not identify an increase in central arterial stiffness among individuals with insulin sensitive prediabetes. Furthermore, no correlation was found between arterial stiffness and fasting glucose measurements. Our data suggest that insulin resistance may be more important in the development of central arterial stiffness than accumulation of AGEs.

The mechanism underlying the relationship between insulin resistance and arterial stiffness is unknown, and current cross-sectional study cannot identify the causative factor. Studies have shown that insulin-resistant states are associated with decreased endothelium-dependent vasodilation [19]. In addition, insulin has been shown to induce vascular smooth muscle proliferation and migration in cell culture [20].

Previous studies have shown that visceral fat in young healthy individuals and older adults is associated with increased central arterial stiffness [18, 21, 22]. The present study confirms this association. In agreement with a recent study by Sabio et al [16], we observed that abdominal adiposity as measured by waist circumference was strongly and adversely associated with aortic stiffness, whereas body mass index as a measure of general adiposity was not. In addition, the present study found that insulin resistance appears to be more strongly associated with arterial stiffness than with measures of obesity. These results suggest that the obesity-arterial stiffness relationship may be mediated in part through increasing insulin resistance. In general, adipocytes, in particular from visceral abdominal regions, produce several bioactive peptides which in turn impact on vascular structure and function [23, 24].

However, increased distending pressure tends to reduce the elasticity of a given arterial segment through the recruitment of collagen fibers [25, 26]. Nevertheless, in our study, as all subjects were normotensive, such the association was not held.

We should point out that the total lipids had insignificant correlation with aortic stiffness. When the components of the lipids were considered separately, aortic stiffness showed direct associations only with triglycerides

In current study, age was an independent predictor of aortic stiffness. It is known that different arterial segments respond differently to aging. Aorta, an elastic artery, loses its compliance with advancing age, whereas compliance in the peripheral, predominantly muscular, arteries appears less closely related to age [15, 27, 28].

In conclusion, we have shown that prediabetic subjects have an aortic stiffness which represent pattern of cardiovascular risk factors, and these changes are predominantly observed in prediabetic subjects with increased HOMA IR and visceral obesity. As the aortic elasticity changes in the prediabetic state are limited to subjects with insulin resistance, the use of insulin-sensitizing agents to prevent diabetes could have a beneficial effect on CVD. None of the studied pre diabetic adult had cardiac complications. The data of the current study report that, as changes can be detected before the appearance of clinically apparent vascular disease, arterial stiffness may act as a marker for the development of future CVD. It is likely that over the next few years measurement of arterial stiffness will become an increasingly important part of the process of risk assessment, and may possibly also improve the monitoring of therapy [29]. Additional studies will be needed to assess the effect of an improvement in insulin sensitivity to decrease arterial stiffness.

It is important to note several limitations in this study design. First, the sample size was relatively small. Second, this study enrolled a relatively population of older individuals carefully screened to exclude diabetes and hypertension. Further research will be needed to confirm whether these results generalize to middle-aged, more insulin-sensitive individuals and those with hypertension through a large community based study.
